# Preparation and Evaluation of Nanocomposite Sodalite/α-Al_2_O_3_ Tubular Membranes for H_2_/CO_2_ Separation

**DOI:** 10.3390/membranes10110312

**Published:** 2020-10-29

**Authors:** Orevaoghene Eterigho-Ikelegbe, Samson O. Bada, Michael O. Daramola

**Affiliations:** 1 DSI-NRF SARChI Clean Coal Technology Research Group, Faculty of Engineering and the Built Environment, University of the Witwatersrand, Wits 2050, Johannesburg, South Africa; eterighoo@gmail.com (O.E.-I.); samson.bada@wits.ac.za (S.O.B.); 2 Department of Chemical Engineering, Faculty of Engineering, Built Environment and Information Technology, University of Pretoria, Hatfield 0028, Pretoria, South Africa

**Keywords:** membrane, pore-plugging hydrothermal, H_2_/CO_2_ separation, sodalite

## Abstract

Nanocomposite sodalite/ceramic membranes supported on α-Al_2_O_3_ tubular support were prepared via the pore-plugging hydrothermal (PPH) synthesis protocol using one interruption and two interruption steps. In parallel, thin-film membranes were prepared via the direct hydrothermal synthesis technique. The as-synthesized membranes were evaluated for H_2_/CO_2_ separation in the context of pre-combustion CO_2_ capture. Scanning electron microscopy (SEM) was used to check the surface morphology while x-ray diffraction (XRD) was used to check the crystallinity of the sodalite crystals and as-synthesized membranes. Single gas permeation of H_2_, CO_2_, N_2_ and mixture gas H_2_/CO_2_ was used to probe the quality of the membranes. Gas permeation results revealed nanocomposite membrane prepared via the PPH synthesis protocols using two interruption steps displayed the best performance. This was attributed to the enhanced pore-plugging effect of sodalite crystals in the pores of the support after the second interruption step. The nanocomposite membrane displayed H_2_ permeance of 7.97 × 10^−7^ mol·s^−1^·m^−2^·Pa^−1^ at 100 °C and 0.48 MPa feed pressure with an ideal selectivity of 8.76. Regarding H_2_/CO_2_ mixture, the H_2_ permeance reduced from 8.03 × 10^−7^ mol·s^−1^·m^−2^·Pa^−1^ to 1.06 × 10^−7^ mol·s^−1^·m^−2^·Pa^−1^ at 25 °C and feed pressure of 0.18 MPa. In the presence of CO_2_, selectivity of the nanocomposite membrane reduced to 4.24.

## 1. Introduction

The application of inorganic membranes for liquid separation and evaporation have proven to be energy-efficient and have demonstrated good stability in the presence of water [1,2,3,4]. According to Li et al. [5], inorganic membranes have the potential to invade territories currently occupied by existing technology like absorption. Hence, these class of membranes are attracting great interest as molecular sieves membranes and are therefore being explored and developed for gas separations. Inorganic membranes are well adapted and promising to perform intrinsic difficult separation such as pre-combustion CO_2_ capture (H_2_/CO_2_ separation) [6,7,8]. Ultimately, the industrial application of inorganic membranes for gas separation in an integrated gasification combined cycle plants could fast track the goal of process intensification in such plants.

Zeolite membranes prepared by growing a thin selective zeolite layer referred to as “thin-film” on top of a support via the direct hydrothermal synthesis method [9,10,11,12,13] and via the secondary-seeded growth method [14,15,16,17] are well reported and have produced good membranes. However, when membranes prepared by these techniques are subjected to large temperature changes, thermal expansion mismatch between the membrane layer and support often leads to grain boundary opening and build-up of long-range stresses [18,19]. As a result, non-selective transport pathways for permeating molecules are created which impacts the performance of the membrane negatively [20,21,22]. To overcome this shortcoming, the pore-plugging hydrothermal (PPH) synthesis was proposed to synthesize quality zeolite membranes [23,24]. In this technique, the zeolite particles are embedded within the pores of the support by temporarily withdrawing the autoclave from the oven during synthesis. By so doing, the expansion of crystals is limited to the pore size of the support [19,25,26,27]. Julbe et al. [28] and Li et al. [23] acknowledged that although, the internal layer of an infiltrated support could contribute to resisting gas transport, the nanocomposite layer is not susceptible to defects, has higher thermal shock resistance, can provide improved membrane separation performance compared to thin-film or surface layer membrane. These qualities are highly attractive for applications in high-temperature environments such as the case in pre-combustion CO_2_ capture.

**Figure 1:**
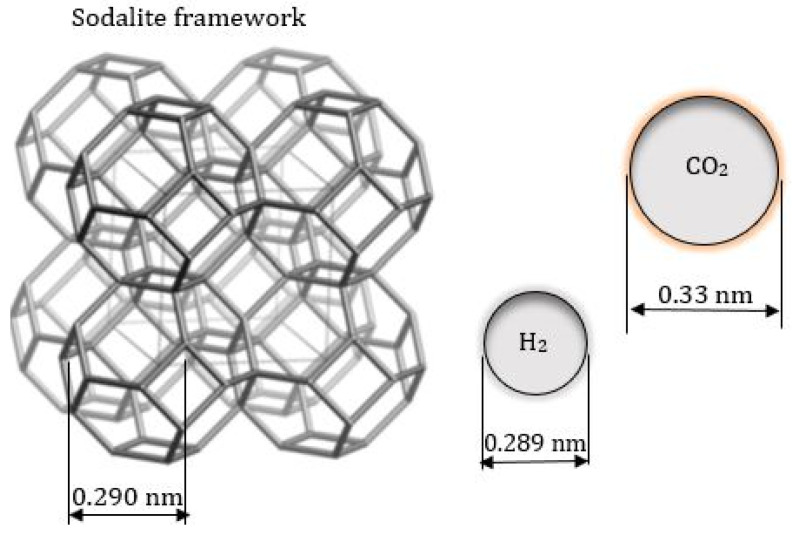
Relative size of SOD structure compared to the kinetic diameter of H_2_ and CO_2_ molecule. HSOD structure adapted from Treacy and Higgins [32] and H_2_ and CO_2_ diameter from Jansen et al. [33] and Xu et al. [34].

Hydroxy sodalite (HSOD) zeolite belongs to the sodalite family and it is formed by connecting sodalite (SOD) cages through common 4- and 6-ring [29,30,31]. The concept for the mechanism of separation of H_2_ from H_2_/CO_2_ mixture using sodalite membrane is highlighted by the size of the SOD aperture relative to the kinetic diameter of H_2_ and CO_2_ shown in Figure 1.

According to Daramola et al. [35], the challenge with developing zeolite membranes via the PPH technique is to reproducibly synthesize high-quality, defect-free supported membranes with high permeance while maintaining high H_2_/CO_2_ selectivity. Also, the number of reports on supported sodalite membranes prepared via the PPH technique focusing on gas separation especially H_2_/CO_2_ separation is still very limited. Daramola et al. [36,37] synthesized nanocomposite hydroxy sodalite (HSOD) ceramic membranes via the PPH technique but the gas permeation measurement and capability of the membrane to sieve molecules was not investigated. Oloye et al. [38] reported the procedure developed by Daramola et al. [36,37] in preparing nanocomposite sodalite (SOD)/ceramic membrane via one-stage and two-stage PPH synthesis. The authors reported SOD fully plugged the 200 nm layer of the α-alumina ceramic support and the nanocomposite SOD/ceramic membrane prepared was moderately selective towards hydrogen as confirmed by SEM. However, the authors did not conduct mixture gas separation test, hence, no information about the real selectivity of the membrane was reported. Recently, Eterigho-Ikelegbe et al. [39] optimized the pore-plugging hydrothermal synthesis to produce quality zeolite crystals. Results revealed zeolite crystals produced using two interruption steps produced the highest quality crystals as confirmed by XRD, SEM, and FTIR.

Therefore, it seems justified to investigate the molecular sieving capability of SOD membrane prepared via the pore-plugging hydrothermal protocol using one interruption step and two interruption steps as reported in this study. Thin-film membranes synthesized via direct hydrothermal synthesis method (Teflon around the outer surface of the support) were also synthesized to compare results. Pure gas of H_2_, CO_2_, and mixture gas of H_2_/CO_2_ was used as evaluation criteria.

## 2. Materials and Methods

### 2.1. Materials

Anhydrous sodium aluminate (NaAl_2_O_3_), sodium metasilicate (Na_2_SiO_3_), and anhydrous sodium hydroxide (NaOH) were used as chemicals for the synthesis of sodalite (SOD) membrane. The chemicals were purchased from Sigma-Aldrich (Pty) (Modderfontein, South Africa) and used without further purification. Deionized water used in the synthesis was prepared in-house and the porous α-alumina (Al_2_O_3_) tubular supports was used without modifications. The tubular supports have an internal diameter of 7 mm and an external diameter of 10 mm with both ends coated with 1 mm non-porous surface for sealing in the module, making the total permeating length 13 cm.

### 2.2. Preparation of SOD Membranes

**Figure 2:**
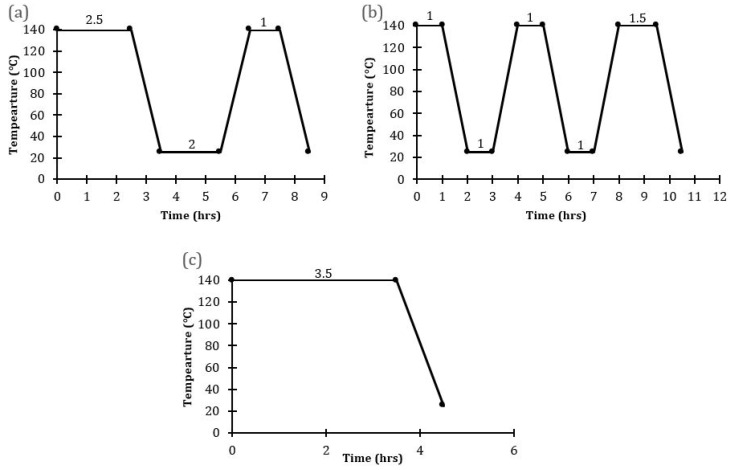
Temperature profiles employed for the synthesis of SOD/α-Al_2_O_3_ (**a**) PPH with one interruption (Membranes M1, M2) (**b**) PPH with two interruptions (Membranes M3, M4) (**c**) direct hydrothermal synthesis (Membranes B1, B2).

SOD membranes were prepared following a modified method of Miachon et al. [24] as reported by Daramola et al. [36]. A precursor solution of molar composition 5SiO_2_:0.5Al_2_O_3_:50Na_2_O:1005H_2_O and a pH of 14 was subjected to the pre-programmed temperature profiles shown in Figure 2. At the end of each synthesis, the hot autoclave was cooled under tap water to obtain the as-synthesized SOD membranes and SOD crystals formed at the bottom of the autoclave. Afterward, the membranes and crystals were washed using deionized water until the pH of the filtrate was 7, then dried at 100 °C overnight in an oven. Two membranes were synthesized for each hydrothermal synthesis protocol.

### 2.3. Membrane Characterization

The crystallinity and purity of the as-synthesized SOD crystals and membranes were checked using x-ray diffractometer (XRD) (Bruker D2 phaser diffractometer equipped with Cu Kα radiation, λ = 1.54184 Å in the 2-theta range of 10–50 °C with a counting step of 31.4 s per step). A scanning electron microscope (FEI NovaLab 600 SEM, Johannesburg, South Africa) was used to check the morphology of the as-synthesized crystals and membrane surface.

### 2.4. Gas Permeation Measurements

**Figure 3:**
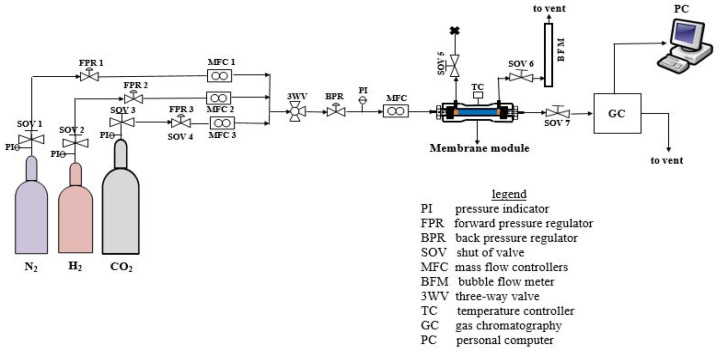
Process Flow Diagram (PFD) of the permeation set-up employed for single and mixture gas permeation experiment.

The as-synthesized SOD membrane quality was evaluated using single gas (H_2_, N_2_, and CO_2_) permeation measurement and H_2_/CO_2_ binary mixtures (H_2_:CO_2_ = 60:40). The mixture ratio was chosen because it represents the H_2_ concentration expected in coal gasification after the water-gas-shift reaction. Before the permeation test, the membranes were thermally treated to a temperature of 100 °C to remove any absorbed moisture and other contaminants. The as-synthesized SOD/ceramic membrane was then gently placed inside a membrane module and held firm in the module using graphite O-rings seals, which also sealed the non-porous ends of the ceramic tube membrane to the membrane module. After setting the laboratory gas supplies from the cylinder to the desired pressure, forward pressure regulators were adjusted to tune individual gas inlet pressure. Thereafter, feed gas was fed to the tube side of the membrane in the module at a specific flow rate, pressure, and temperature. Permeate gas flowed through the shell side of the membrane for both single and mixture gas experiments. The test was conducted without sweep and permeate side pressure was kept at atmospheric pressure all through. The temperature of the feed gas was increased in small increments to avoid sudden overheating of the furnace and module. Permeation was measured as a function of pressure ranging from 0.1 MPa to 0.5 MPa and at a temperature ranging from 25 °C to 200 °C in the dead-end mode (i.e., retentate stream blocked) for single gas experiments. During the mixture gas experiment, the retentate stream (SOV 7) was left opened and controlled by a valve, the permeate stream was vented to the atmosphere, while SOV 5 remained locked (see Figure 3). To obtain consistency in data reporting under a set of experimental variables, at least one-hour equilibrium time was necessary for the system to reach steady-state. Also, the experimental test for each membrane was done twice to ensure reliable statistical average. A Bruker 430-GC gas chromatography equipped with a thermal conductivity detector (TCD) and coupled with stainless-steel column (2 m, OD:3.175 mm, ID:2 mm) was used to analyze the composition of the permeate streams. The gas composition in the retentate stream was not known, however, a mass balance was used to get retentate composition. Permeance П_i_, ideal selectivity (*S_i/j_*), separation factor (*SF_i/j_*) was obtained using Equations (1)–(3), respectively.

(1) $${П}_{i}\;=\;\frac{F_{i}}{∆P_{i}}\;=\;\frac{f_{i}}{A\;\times \;∆P_{i}}\;[\mathrm{mol}\cdot s^{-1}\cdot m^{2}\cdot {\mathrm{Pa}}^{-1}]$$

(2) Sij = ПiПj [-]

(3) SFij=(yiyj)permate(xixj)feed         [-]

where *F_i_* is the flux of specie *i* through the membrane, *f_i_* is the molar flow rate of the gas, *A* is membrane, *P_i,f_* and *P_i,p_* are pressures of component *i* in the feed, and permeate sides, respectively, Δ*P_i_* is the trans-membrane pressure across the membrane of a gas component *i*, ${П}_{i}$ and ${П}_{j}$ are the permeance of pure gas feed *i* (H_2_) and feed *j* (CO_2_), respectively, *y* and *x* are the mole fraction of gas components on the permeate and feed side of the membrane.

## 3. Results and Discussion

### 3.1. Membrane Characterization

**Figure 4:**
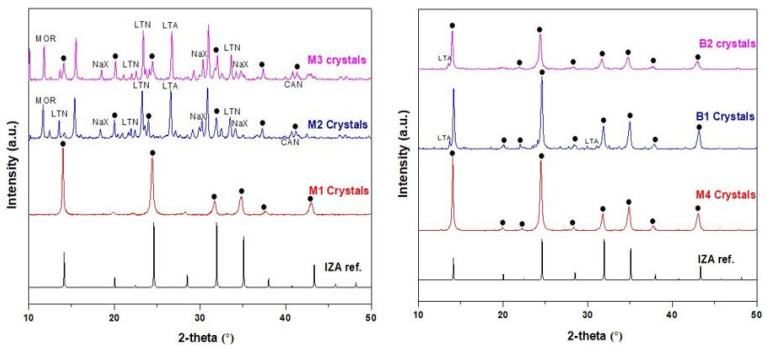
XRD patterns of the crystals obtained from the bottom of the autoclave during the synthesis of membranes M1, M2, M3, M4, B1, and B2. [(•) represents sodalite peak].

Sodalite crystals were recovered from the bottom of the autoclave after each of the membrane synthesis and were examined to confirm the purity of the SOD crystals that made the membrane via XRD analysis. The XRD patterns of SOD crystals obtained from M1 and M4 membrane depicted in Figure 4 reveal that the patterns of the synthesized SOD agree well with that of the simulated XRD pattern of SOD from the International Zeolite Association (IZA) [32], and also agree with other synthesized SOD crystals from literature [9,28,39,40,41,42]. Based on these observations, it is clear that the SOD crystals grown within the alumina to form the membrane are pure SOD crystals. On the other hand, the XRD patterns of SOD crystals obtained for membranes M2 and M3 contain some impurities which are other types of zeolites such as LTN, NaX, CAN, LTA, MOR. The crystallization of larger size zeolites led to higher porosity as will be discussed in Section 3.2. The presence of impurity phases i.e., phases that are not sodalite indicates qualitatively that membranes M2 and M3 were of very low quality. Very weak LTA peaks were observed on the XRD patterns of membrane B1 and B2, which might have been as a result of external influences in the laboratory they were not easy to control.

**Figure 5:**
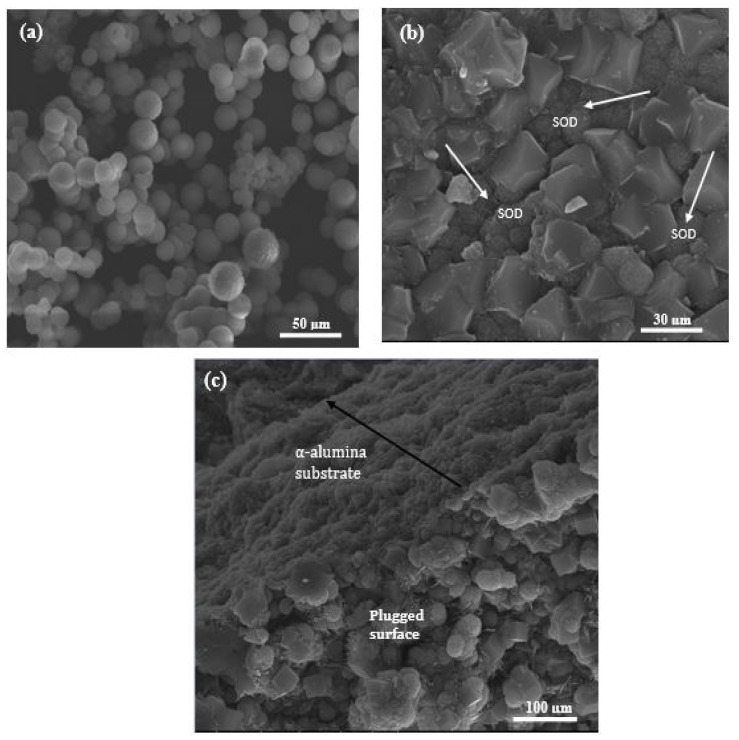
SEM micrographs of (**a**) SOD crystals collected from the bottom of the autoclave, (**b**) membrane surface, and (**c**) membrane cross-section prepared via the pore-plugging hydrothermal synthesis method.

**Figure 6:**
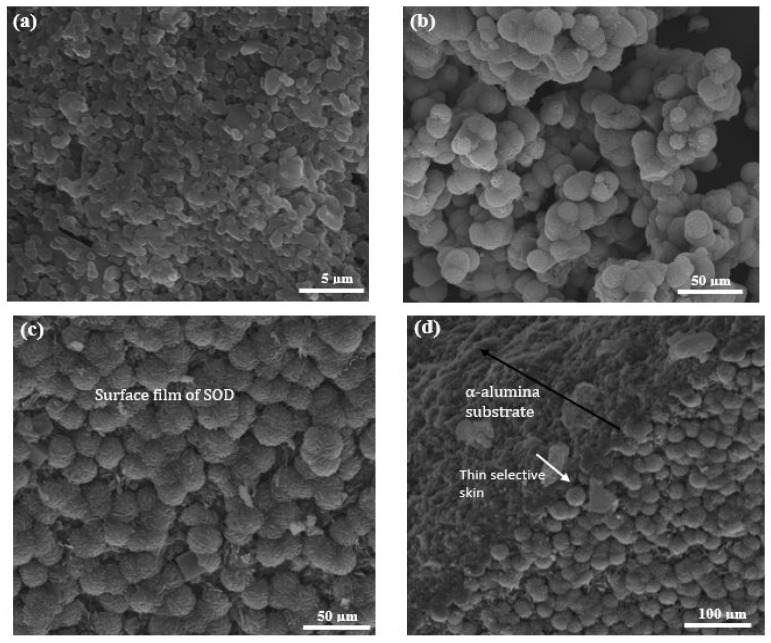
SEM images of (**a**) 200 nm layer (Innermost layer) of the α-Al_2_O_3_ support; (**b**) SOD crystals obtained from the bottom of the autoclave during PPH, (**c**) surface of membrane prepared via the direct hydrothermal synthesis, and (**d**) cross-section of membrane prepared via the direct hydrothermal synthesis.

The SEM micrographs of the as-synthesized membranes prepared via PPH and via direct hydrothermal synthesis are presented in Figure 5 and Figure 6. From the micrograph (Figure 5b), it can be observed that SOD crystals were indeed deposited within the inner surface pores of the support. However, one cannot rule out the formation of incomplete pore-plugging within the middle layer of the support (800 nm layer) as this could not be confirmed from the SEM images. Figure 5c displays the cross-section of the as-synthesized nanocomposite SOD/ceramic membrane showing a continuous separative layer and the pore-plugged cross-section. In nanocomposite membranes, there is a change in temperature which causes a change in the autogenous pressure within the autoclave. This change brought about by the interruption might have facilitated flow of precursor solution into the pore of the support, thereby resulting in the growth of SOD crystals that form the separative layer within the pores [36,37]. The formation of the separative zeolite layer within the pores of the ceramic support via pore plugging method controls the formation of defects and limits the growth of the crystals within the size of the pores. In addition, having the separative layer within the pores of the ceramic support protects it from abrasion that could occur during membrane handling and also against thermal-induced defects due to thermal shocks when compared to the thin film counterparts [24,26].

Membrane prepared via the direct hydrothermal synthesis technique also shows a continuous growth of SOD crystals on the innermost layer of the support (200 nm layer), though with a few defects (see Figure 6c). It is noteworthy to mention that the 1200 nm layer (outermost layer) of the support was completely wrapped with Teflon tape to prevent penetration of precursor solution into the pores of the support during the direct hydrothermal synthesis. As a comparison, SEM image of the ceramic support (tube side) is shown in Figure 6a. From the micrograph depicted in Figure 6d, a clear interface between the zeolite layer and support could be identified with limited penetration of the zeolitic layer into the underlying pores of the support. These observations confirm that a thin-film membrane was formed, and also agrees with similar studies conducted by Fan et al. [41] and Lafleur et al. [27]. Micrographs of SOD crystals collected from the bottom of the autoclave after the hydrothermal synthesis are depicted in Figure 5a and Figure 6b. The SEM images in Figure 5a and Figure 6a show thread-ball shapes of SOD crystals and this observation agree with literature [15,27,28,39,41].

### 3.2. Single Gas Permeation

Comparison of results obtained from the single gas permeation experiments provide information about the reproducibility of each technique for the synthesis of the membrane. A summary of the results from the single gas permeation conducted at room temperature (25 °C) and at 0.18 MPa feed pressure is presented in Table 1.

H_2_ permeance of the membranes M1, M4, B1, and B2 were the highest compared to that of N_2_ and CO_2_. This was expected as H_2_ possesses the smallest kinetic diameter of 0.289 nm. Membrane M2 and M3 displayed similar H_2_ and CO_2_ permeance. This is abnormal considering the size of the CO_2_ molecule (0.33 nm) to the pore dimension of SOD (0.29 nm). From the XRD patterns (Figure 4), the presence of large pore zeolitic phases such as Na-A (0.41 nm), LTA (0.3-0.45 nm), Na-X (0.73 nm) type zeolites were formed alongside SOD zeolite. The presence of these zeolites in the membranes could have contributed to the relative high CO_2_ permeance recorded. Even these zeolites could have been instrumental to the surface roughness or even poor intergrowth observed in the membranes. As mentioned in a previous study [39], the presence of different type of zeolites as impurities inside the pores of the support could result in many intercrystalline defects. As a result, CO_2_ permeance from the synthesized membranes, M2 and M3, in this study were unexpected. Based on these observations, membranes M2 and M3 were considered in the further investigations as reported in this article. The single permeation results for H_2_, N_2_ and CO_2_ from B1 and B2 (membranes prepared via direct hydrothermal synthesis) were in the order of H_2_ > N_2_ > CO_2_. Since the kinetic diameters of these gases are 0.28 nm for H_2_, 0.36 nm for N_2_ and 0.33 nm for CO_2_, one would expect the order as H_2_ > CO_2_ > N_2_. Thus, the observed order could be attributed to the presence of other zeolites as impurities in the membranes as observed in the membranes prepared via PPH. However, membranes prepared via PPH synthesis (M1, M4) show a reverse trend i.e., permeance reduced as kinetic diameter increased (H_2_ > CO_2_ > N_2_). The observed trend could support the hypothesis that gas permeation through membranes prepared by the PPH technique was controlled by the molecular sieving through zeolite channels rather than surface diffusion through grain boundaries as reported in elsewhere [34,43,44].

#### 3.2.1. Effect of Temperature

**Figure 7:**
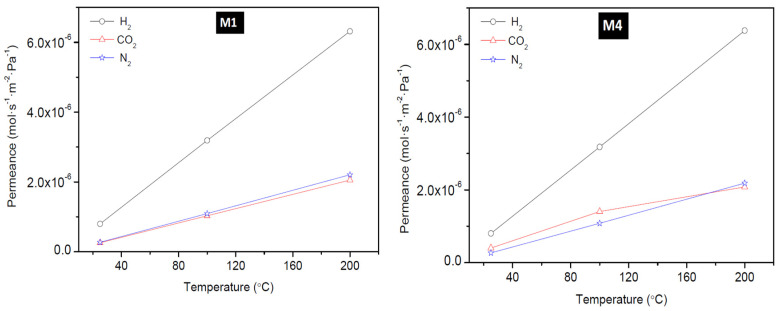
Effect of permeation temperature on H_2_, CO_2_, and N_2_ permeance of membrane M1 (PPH-1 interruption) and M4 (PPH-2 interruptions).

**Figure 8:**
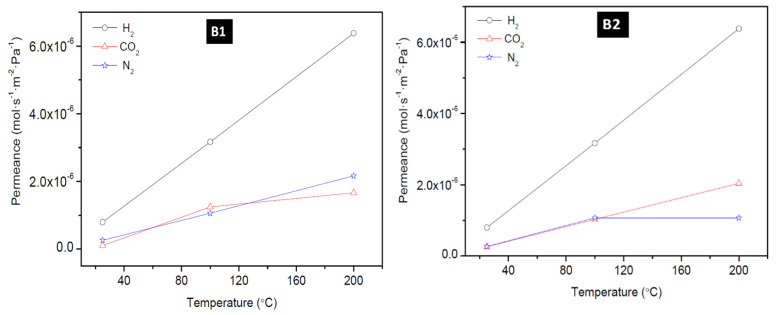
Effect of permeation temperature on H_2_, CO_2_, and N_2_ permeance of membrane B1 and B2 prepared via the direct hydrothermal method.

To have a better understanding of the performance of the as-synthesized membranes, the temperature dependence of the transport property of the membranes was investigated. The effect of temperature on the permeation of H_2_, CO_2_, and N_2_ at a feed pressure of 0.18 MPa through the membrane: M1, M4, B1, and B2 is presented in Figure 7 (for M1 and M4,) and Figure 8 (for B1 and B2). These figures show that H_2_ permeance exhibited a strong temperature dependence. Though this behaviour considers the mechanism of the permeation behaviour in the context of the single gas permeation behavior with temperature as reported elsewhere [22,45]. Thus, it is evident that at low temperature and at ambient pressure, H_2_ was only weakly absorbed on SOD zeolite as seen from Figure 7 and Figure 8. It was observed also that the permeance of H_2_ through the SOD membrane increased with increasing temperature, indicating that H_2_ permeation through the membranes could be governed by activated diffusion behavior [46].

Also, H_2_ permeance does not show a maximum in the range of temperature tested as reported elsewhere. For example, a study by Kanezashi and Lin [47] where H_2_ and CO_2_ permeation through MFI membranes synthesized on alumina support via secondary seeded growth method was measured shows that the permeance of H_2_ and CO_2_ decreased with increasing temperature. The decrease in permeance was attributed to the Knudsen-type temperature dependency display of the membrane up to 500 °C and this agrees with studies reported elsewhere [48,49,50]. At the same time, these studies [48,49,50] suggested that increase in H_2_ and CO_2_ permeance with an increase temperature indicates that permeation is controlled by activated diffusion instead by Knudsen diffusion. On the other hand, constant CO_2_ permeance at increasing temperature observed by Farjoo and coworker [51] was attributed to the combined contributions from activated process and non-zeolitic flux.

#### 3.2.2. Effect of Feed Pressure

**Figure 9:**
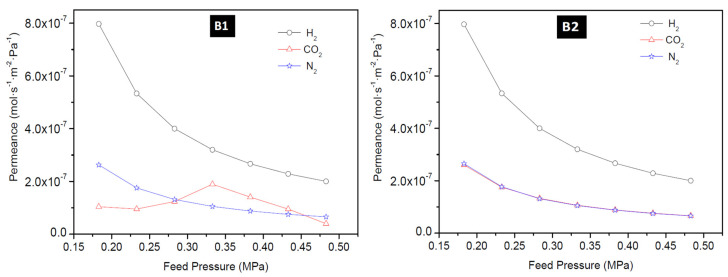
H_2_ permeance, CO_2_ permeance and N_2_ permeance as a function of feed pressure at 25 °C for membrane M1 prepared using PPH (1-interruption) and M4 using PPH (2-interruptions).

**Figure 10:**
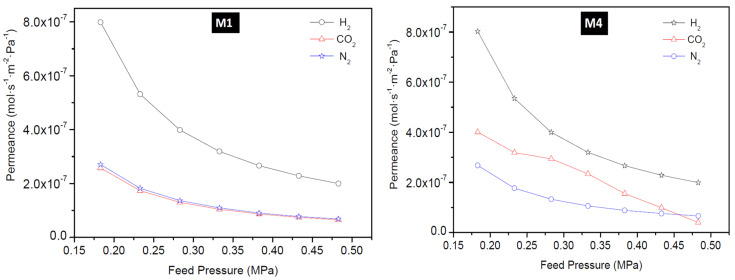
H_2_ permeance, CO_2_ permeance, and N_2_ permeance as a function of feed pressure at 25 °C for membrane B1 and B2 prepared using the direct hydrothermal method.

The permeance of H_2_, CO_2_, and N_2_ as a function of feed pressure at 25 °C for membrane M1, M4, B1, and B2 is presented in Figure 9 and Figure 10. The permeance of H_2_, CO_2_, and N_2_ were observed to depend on the feed pressure for the temperature range considered in this study. Maximum H_2_ permeance obtained for membrane M1 was 6.32 × 10^−6^ mol·s^−1^·m^−2^·Pa^−1^ at 200 °C and 0.18 MPa feed pressure and this value reduced to 1.58 × 10^−6^ mol·s^−1^·m^−2^·Pa^−1^ when the pressure was increased. Similarly, CO_2_ permeance and N_2_ permeance decreased as the pressure was increased. This is expected because permeance is inversely proportional to the change in transmembrane pressure. Since the pressure on the permeate side was kept constant during the experiment, an increase in the feed pressure indicates an increase in the transmembrane pressure, thus a decrease in the permeance. The lowest N_2_ permeance and CO_2_ permeance obtained for membrane M1 at room temperature (25 °C) and 0.48 MPa were 6.83 × 10^−8^ mol·s^−1^·m^−2^·Pa^−1^ and 6.50 × 10^−8^ mol·s^−1^·m^−2^·Pa^−1^, respectively. According to Hosseinzadeh Hejazi et al. [50], a constant permeance or a slight reduction in permeance as a function of pressure should be observed if the zeolitic pores are greater than the kinetic diameter of the permeated molecules and the permeation is only through the zeolitic pores. Gas can permeate through zeolitic pores and non-zeolitic pores in zeolite membranes. When these defects (non-zeolitic pores) are relatively large, they provide non-selective pathways, and viscous flow becomes the predominant mechanism with an increase in pressure. Also, presence of small defects will result in Knudsen mechanism controlling the separation, thus making the membrane semi-selective with flux remaining almost constant as pressure increased [50,51]. Constant flux at increasing feed pressure, while keeping the permeate pressure constant, will result in the decrease of permeance as a function of pressure as observed in this study and could indicate presence of defects in the membranes as well. The effect of temperature on H_2_ permeation of HSOD zeolite membrane investigated by Vaezi and Babaluo, [10] showed that H_2_ permeance and CO_2_ permeance slightly increased as a function of mean pressure and this was attributed to the presence of impurities providing larger pore sizes than the size of the HSOD membranes for the transport of smaller gases.

#### 3.2.3. Ideal Selectivity

**Figure 11:**
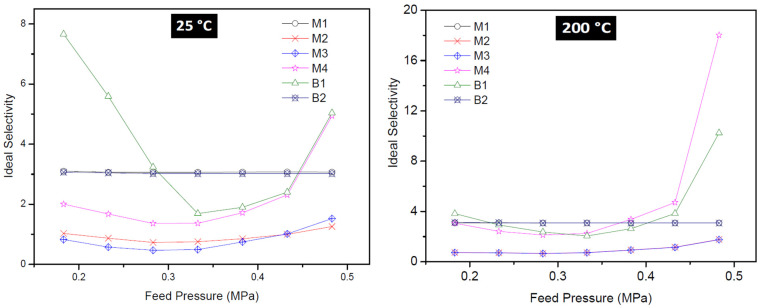
Ideal selectivity of H_2_/CO_2_ as a function of feed pressure of all membranes at 25 °C (**left**) and 200 °C (**right**).

Ideal selectivity during the single gas permeation was obtained for the gases and all the as-synthesized membranes in this study. The ideal selectivity of H_2_/CO_2_ as a function of feed pressure for all the membranes is presented in Figure 11. Membrane M1 and B2 displayed a near-constant ideal selectivity of 3.10 for the range of feed pressure investigated at 25 °C. This value is lower than the predicted theoretical H_2_/CO_2_ Knudsen selectivity of 4.7, suggesting little contribution of zeolitic pores to the permeation in these two membranes. Even, the selectivity of these membranes did not improve when the temperature was increased to 200 °C. Membrane M2 and M3 displayed the least ideal selectivity value of 1.52 which supported the early conclusion that these membranes were of very poor quality because of the presence of other phase zeolites.

At high temperatures, CO_2_ adsorption on the surface of the zeolite pores of M2 and M3 weakened dramatically and its mobility increased. As a result, membranes M2 and M3 displayed the least H_2_/CO_2_ selectivity (ideal). In other words, these membranes did not show molecular sieving ability. Instead the membranes possess large non-selective pores that are larger than the kinetic diameter of H_2_ and CO_2_, thereby allowing viscous flow through the pores of these membranes.

Membrane B1 displayed high H_2_/CO_2_ selectivity (ideal) of 7.66 at 0.18 MPa and 10.25 at 0.48 MPa, indicating more contribution of the zeolitic pores to the permeation and also that a drastic decrease in the number of non-zeolitic/non-selective pores whose average size is greater than the kinetic diameter of the permeated gases. Membrane prepared via the pore-plugging hydrothermal synthesis using two interruptions steps of one hour each (membrane M4) resulted in membranes displaying the best quality and thus the best-performing membrane. The membrane displayed ideal selectivity for H_2_/CO_2_ of 4.94 at 25 °C and 18.03 at 200 °C, at a feed pressure of 0.48 MPa. The high selectivity can be attributed to the much stronger adsorption of CO_2_ on the membrane surface which limits its diffusion through the membrane. By interrupting the synthesis two times, more precursor would have transported inside the pores of the support leading to complete blocking of the support pores by sodalite crystals after the second interruption. Based on these findings, it can be concluded that the fraction of “non-selective” viscous flux in membrane M4 was very small. Hence, membrane M4 possesses more zeolitic pores that contribute to selective separation with very little non-zeolitic pores. It is noteworthy to mention that a decrease in ideal selectivity with increasing pressure suggests gas transport flow through non-zeolitic regions and macroporous defects (viscous flow contribution) [28,50].

### 3.3. Mixture Separation Test

**Figure 12:**
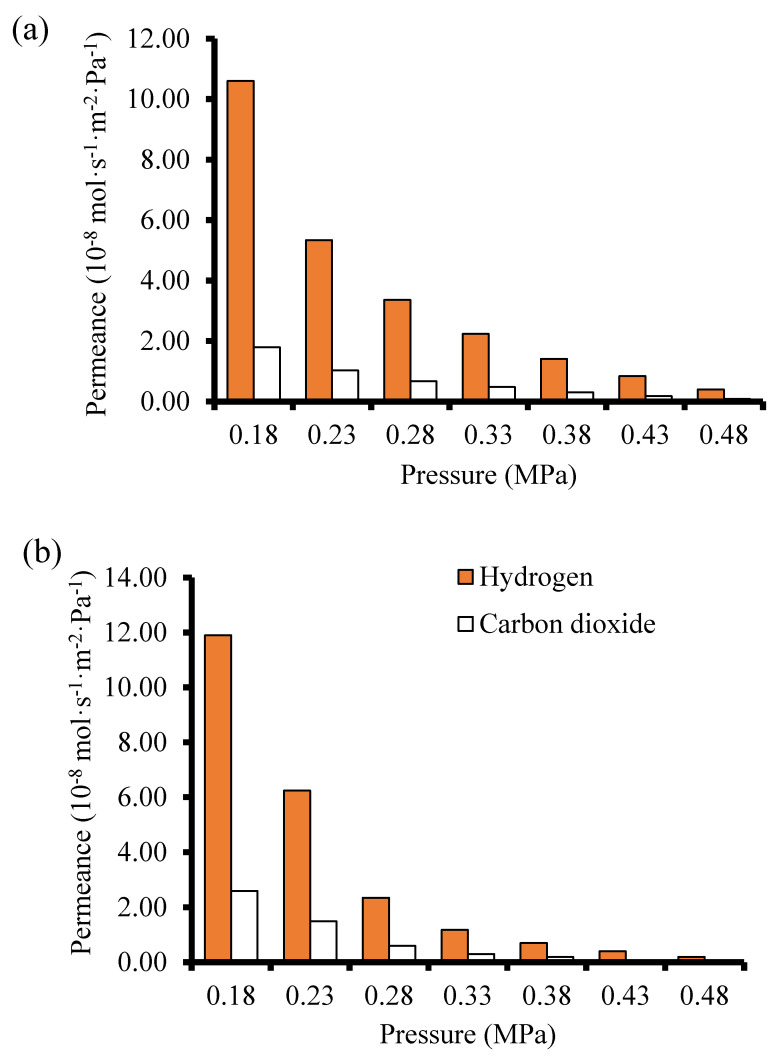
Gas permeance as a function of pressure during the mixture gas separation at 25 °C (**a**) membrane M4 prepared using 2-interruption steps PPH synthesis method (**b**) Membrane B1 prepared by the direct hydrothermal synthesis method.

Membranes M4 and B1 that displayed the best single gas permeation results (considering the H_2_/CO_2_ ideal selectivity) were tested in H_2_/CO_2_ (60/40) mixture separation. During the mixture gas separation, H_2_ permeance obtained was 1.19 × 10^−7^ mol·s^−1^·m^−2^·Pa^−1^ for membrane B1 and 1.06 × 10^−7^ mol·s^−1^·m^−2^·Pa^−1^ for membrane M4 at 25 °C and 0.18 MPa feed pressure. From Figure 12, the permeance of the weakly adsorbed H_2_ is more reduced when compared to that of the strongly adsorbing CO_2_. According to Lindmark and Hedlund [52], this suggests that the effective pore size of the membrane prepared in this study approaches the size of the permeating molecules. The H_2_ permeance and the CO_2_ permeance observed at the same temperature, 25 °C, for both single gas permeation experiments and the mixture gas separation experiments show that H_2_ permeance in the single gas permeation is 6–7 times lower that the value obtained during the mixture gas separation experiments. This observation could be attributed to the competition between the two gases during the mixture gas separation due to adsorbate-adsorbate interaction and/or adsorbate-membrane wall interaction.

Table 2 shows the comparison of the H_2_/CO_2_ separation performance of the membranes fabricated and tested in this study with literature. Though the results obtained from literature employed different type of membranes, the differences in the separation performance of membranes synthesized in this study compared to that of the membranes from literature could be attributed to the difference in preparation protocols and the membrane supports. Membrane M4 prepared via pore-plugging hydrothermal synthesis technique using two interruption steps displayed a higher separation factor (SF) of 4.24 for H_2_/CO_2_ mixture at a high feed pressure of 0.48 MPa and at 25 °C. The SF increased as feed pressure increased from 0.18 MPa to 0.48 MPa. The SF for membranes prepared in this study tested at 25 °C is lower than the SF of 7.2 for the H_2_/CO_2_ at 50 °C for ZIF 22 reported by Huang et al. [53]. The pore size of ZIF 22 was about 0.3 nm, therefore it is expected that its separation performance for H_2_/CO_2_ will be higher when compared to that of the membranes in this study. It is noteworthy to mention that a binary mixture of H_2_/CO_2_ (50:50) was used in ref. [53] instead of 60:40 used in this work was used. Though the partial pressure of the feed used in this work is higher than the one used in ref. [53], but conducting the separation reported in ref. [53] at Wicke–Kallenbach mode where TMP = 0 and with the use of a sweep gas might contribute to the higher performance reported. Furthermore, Yin et al. [54] prepared zeolite NaX composite membrane via the secondary seeded growth method and employed the membrane to separate a 50:50 mixture of H_2_/CO_2_. The authors obtain a SF of 4.57. NaX is a zeolite with polar sites and has strong electrostatic interactions with polar gas like CO_2_ which contributed to H_2_/CO_2_ separation. SAPO-34 zeolite membrane prepared on chitosan modified support for H_2_/CO_2_ separation was reported by Das et al. [55]. The authors reported that the SF for H_2_/CO_2_ increased to 4.2 when the partial pressure of CO_2_ in the feed was increased. By synthesizing and using a unique membrane having a bilayer membrane structure with an intermediate macroporous yttria stabilized zirconia (YSZ) for H_2_/CO_2_ separation, Wang et al. [56] reported a H_2_/CO_2_ SF of 25.3 at 450 °C. Huang and co-workers [14] prepared LTA zeolite membranes in the presence of covalent linkers via the direct hydrothermal synthesis method on disk support and reported a SF of 5.5 and CO_2_ permeance of 6.8 × 10^−8^ mol·s^−1^·m^−2^·Pa^−1^ for the membrane when used to separate H_2_/CO_2_ mixture.

Considering the different experimental conditions, the different support used, and the different synthesis techniques employed by these authors, the SF for H_2_/CO_2_ obtained for SOD membrane (4.13–4.24) in this study is very comparable to values reported in literature, though still lower than the theoretical value of Knudsen diffusion of 4.7. Based on these findings, it is likely that the gases permeated through the grain boundary in the membranes synthesized in this study rather than through the zeolitic pores of the SOD. However, interrupting the hydrothermal synthesis twice had a positive effect as mentioned in Section 3.2.2. By synthesizing zeolite membrane without interruption, zeolite crystals are only grown on the surface of the membranes. On the other hand, interrupting the synthesis only once may have led to inefficient pore-plugging of the support with zeolite crystals.

## 4. Conclusions

Nanocomposite SOD/α-alumina membranes, prepared via the pore-plugging hydrothermal (PPH) synthesis using one interruption, two-interruption steps, and prepared via direct hydrothermal synthesis have been presented. These membranes were characterized and tested for separation of H_2_/CO_2_ mixture, first by single gas permeation tests and then by mixture gas separation. The nanocomposite membrane, M4, synthesized via PPH synthesis using two interruption steps displayed the best ideal selectivity of 8.76 compared to the thin-film membranes during the single gas permeation tests. The H_2_ permeance of this membrane was 1.59 × 10^−6^ mol·s^−1^·m^−2^·Pa^−1^ at 200 °C and 0.48 MPa. The good performance of this membrane could be attributed to the due to enhanced pore plugging effect after the second interruption step. When the membrane was tested at room temperature using a binary gas mixture of H_2_/CO_2_ (60:40), the H_2_ permeance decreased to 1.06 × 10^−7^ mol·s^−1^·m^−2^·Pa^−1^ attributable to the adsorbate-adsorbate and adsorbate-membrane wall interactions during the separation. However, the results have demonstrated that by optimizing the synthesis variables during PPH could pave the way for the development of high-quality nanocomposite SOD/α-alumina membranes that could display better separation performance for H_2_/CO_2_ separation. Availability of this type of membranes could fast track the development and commercial the Integrated Gasification Combined Cycle (IGCC) technologies employable in the pre-combustion CO_2_ capture.

## Figures and Tables

**Table 1:** Single gas permeation results of the as-synthesized membranes at 25 °C and 0.18 MPa. PPH: Pore-Plugging Hydrothermal.

Membrane Ref.	Synthesis Technique	H_2_ Permeance	CO_2_ Permeance	N_2_ Permeance
		(10^−7^ mol·s^−1^·m^−2^·Pa^−1^)		
M1	PPH (1-interruption)	7.99	2.58	2.21
M2	PPH (1-interruption)	8.00	7.78	2.91
M3	PPH (2-interruptions)	7.95	9.63	2.71
M4	PPH (2-interruptions)	8.03	4.01	2.68
B1	Direct hydrothermal	7.97	1.04	2.63
B2	Direct hydrothermal	7.97	2.60	2.65

**Table 2:** Comparison of mixture gas separation data of the SOD membrane in this study with other membranes in the literature. W-K: Wicke–Kallenbach technique; DH: Direct hydrothermal; SR: Solvothermal Reaction; PI: Phase Inversion; SG/UI: Seeded Growth/Ultrasonic Irradiation; SSG: Secondary Seeded Growth; PPH: Pore-Plugging Hydrothermal; SSOD/PSF: Silica Sodalite/Polysulfone; SF: Separation Factor.

Zeolite	Support	Preparation Technique	Temp (°C)	TMP (KPa)	H_2_: CO_2_	Permeance (10^−8^ mol·s^−1^·m^−2^·Pa^−1^)		SF	Ref.
						H_2_	CO_2_		
LTA	α-Al_2_O_3_ disk	DH	20	W-K	50:50	30	6.80	5.3	[53]
ZIF-22	TiO_2_ disk	SR	50	100 W-K	50:50	16.6	2.30	7.2	[14]
ZIF-7-NH_2_	α-Al_2_O_3_ disk	SSG	25	W-K, N_2_ sweep	50:50	10	0.6	19	[57]
ZIF-8	γ-/α-Al_2_O_3_ disc	CD	250	W-K, He sweep	50:50	9	-	8.2	[58]
LTA	Clay–Al_2_O_3_	SG/UI	25	300	50:50	-	-	15.3	[59]
ZIF-7	α-Al_2_O_3_ disk	SSG	220	W-K, N_2_ sweep	50:50	4.55	0.33	13.6	[60]
NaX	Stainless-steel net	SSG	16	-	50:50	10.1	-	4.57	[54]
SAPO-34	Clay- Al_2_O_3_ tube	SSG	25	200	70:30	-	-	4.2	[55]
MFI	α-Al_2_O_3_ disk	SSG	100	100	50:50	174	-	1.02	[56]
LTA/AlPO_4_	α-Al_2_O_3_ disk	SSG	20	100	50:50	24	3.17	7.6	[61]
SSOD/PSF	-	PI	25	100	60:40	49.2	42.2	2.2	[62]
SOD (M4)	α-Al_2_O_3_ tube	PPH	25	220	60:40	10.6	1.79	4.24	This study
SOD (B1)	α-Al_2_O_3_ tube	DH	25	220	60:40	11.9	2.55	4.13	This study
